# Diversity of putative archaeal RNA viruses in metagenomic datasets of a yellowstone acidic hot spring

**DOI:** 10.1186/s40064-015-0973-z

**Published:** 2015-04-18

**Authors:** Hongming Wang, Yongxin Yu, Taigang Liu, Yingjie Pan, Shuling Yan, Yongjie Wang

**Affiliations:** College of Food Science and Technology, Shanghai Ocean University, Shanghai, China; Laboratory of Quality and Safety Risk Assessment for Aquatic Products on Storage & Preservation, Ministry of Agriculture, Shanghai, China; Shanghai Engineering Research Center of Aquatic-Product Processing & Preservation, Shanghai, China; College of Information Technology, Shanghai Ocean University, Shanghai, China; Institute of Biochemistry and Molecular Cell Biology, University of Goettingen, Goettingen, Germany

**Keywords:** Putative archaeal RNA viruses, Sequence assembly, Viral diversity, Yellowstone acidic hot spring

## Abstract

Two genomic fragments (5,662 and 1,269 nt in size, GenBank accession no. JQ756122 and JQ756123, respectively) of novel, positive-strand RNA viruses that infect archaea were first discovered in an acidic hot spring in Yellowstone National Park (Bolduc et al., 2012). To investigate the diversity of these newly identified putative archaeal RNA viruses, global metagenomic datasets were searched for sequences that were significantly similar to those of the viruses. A total of 3,757 associated reads were retrieved solely from the Yellowstone datasets and were used to assemble the genomes of the putative archaeal RNA viruses. Nine contigs with lengths ranging from 417 to 5,866 nt were obtained, 4 of which were longer than 2,200 nt; one contig was 204 nt longer than JQ756122, representing the longest genomic sequence of the putative archaeal RNA viruses. These contigs revealed more than 50% sequence similarity to JQ756122 or JQ756123 and may be partial or nearly complete genomes of novel genogroups or genotypes of the putative archaeal RNA viruses. Sequence and phylogenetic analyses indicated that the archaeal RNA viruses are genetically diverse, with at least 3 related viral lineages in the Yellowstone acidic hot spring environment.

**Electronic supplementary material:**

The online version of this article (doi:10.1186/s40064-015-0973-z) contains supplementary material, which is available to authorized users.

## Background

Almost all life forms can be infected by viruses. To date, thousands of viruses have been identified (King et al. 2012). However, most of these viruses infect bacteria or eukaryotes. Compared to the more than 6,000 viruses that infect bacteria (Ackermann 2007; Ackermann and Prangishvili 2012), there are fewer than 100 viruses of archaea (Pina et al. 2011), all of which harbor DNA genomes (Prangishvili 2013).

Viruses in the environment are abundant, and viral communities are incredibly diverse (Breitbart et al. 2002; Breitbart and Rohwer 2005; Angly et al. 2006; Breitbart 2012). There are an average of 10^7^ virus-like particles per milliliter of surface seawater (Bergh et al. 1989), an estimated 5,000 viral genotypes in 200 liters of seawater (Breitbart et al. 2002) and at least 10^4^ viral genotypes in one kilogram of marine sediment (Breitbart et al. 2004). The presence of archaeal RNA viruses in the environment is likely considering both the large number of various RNA viral types infecting eukaryotes and bacteria (Culley et al. 2006; Prangishvili et al. 2006; Lang et al. 2009) and that archaea comprise up to one-third of the ocean’s prokaryotes (Karner et al. 2001).

Recently, sequences of putative archaeal RNA viruses were obtained using a metagenomic approach (Bolduc et al. 2012). Viral samples were collected from high-temperature, acidic hot springs in Yellowstone National Park, and viral RNA was extracted and transcribed into cDNA for metagenomic sequencing. Two contigs were assembled and were demonstrated to be genomes of putative archaeal RNA viruses (GenBank accession no. JQ756122 and JQ756123) (Bolduc et al. 2012).

The nucleotide sequence JQ756122, which is 5,662 nt in length, is thought to be a near-full-length genome of the putative archaeal RNA viruses and contains a single open reading frame that encodes a putative viral polyprotein encompassing an RNA-dependent RNA polymerase and a putative capsid protein (Bolduc et al. 2012). The second sequence, JQ756123, with a length of 1,269 nt, encompasses three overlapping short ORFs, each of which shows approximately 70% amino acid sequence identity with the predicted RNA-dependent RNA polymerase of JQ756122 (Bolduc et al. 2012).

Here, we investigate the genetic diversity of the putative archaeal RNA viruses in global metagenomic datasets based on sequence assembly. Sequence and phylogenetic analyses indicate that at least three lineages of the putative archaeal RNA viruses may be present in Yellowstone hot springs.

## Methods

### Sequence assembly

The nucleotide sequences of the putative archaeal RNA viruses (GenBank accession no. JQ756122) was downloaded from GenBank and was searched (BLASTN, E-value < 10^−5^) against the NCBI non-redundant nucleotide database. Hits with a significant level (E-value < 10^−5^) included those two nucleotide sequences of JQ756122 and JQ756123, which were identified as nucleotide sequences of putative archaeal RNA viruses, suggesting that JQ756122 was archaeal RNA virus-specific and was well conserved, making it easy to map reads in metagenomic databases.

**Figure 1:**
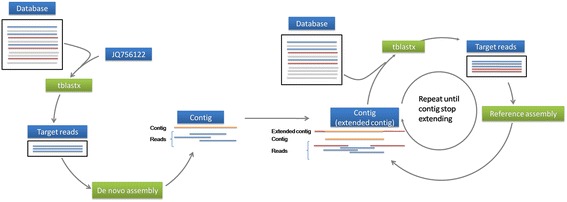
Schematic presentation of the sequence assembly procedures.

Subsequently, JQ756122 was used to search (TBALSTX, E-value < 10^−5^) all of the databases on the CAMERA 2.0 portal (http://camera.calit2.net). Hits were obtained from four databases (Additional file 1: Table S1). The broad phage metagenome database contained the largest number (n = 3,763) of matched reads, including all of the reads that were detected in both the metagenomic 454 whole genome shotgun reads and the metagenomic 454 reads databases (Additional file 1: Table S1). Only one hit, JQ756122, was found by searching the NCBI environmental sample nucleotide database. Subsequently, these 3,763 reads, which had significantly similarity to JQ756122, were downloaded from the CAMERA 2.0 portal (Additional file 1: Table S1) and further analyzed for their RNA source based on information regarding the nucleotide samples. As a result, 6 reads originating from natural DNA samples were removed, while the remaining 3,757 reads of RNA samples (Additional file 2: Table S2) were all from an acidic hot spring in Yellowstone National Park and were used for de novo assembly to obtain JQ756122-related contigs. Each contig was searched separately (TBALSTX, E-value < 10^−5^) against the broad phage metagenome database in the CAMERA 2.0 portal. Reads that were significantly similar to the contig were downloaded from the CAMERA 2.0 portal and checked for RNA origin. The contig then served as a reference sequence to assemble these retrieved reads. Once an extended contig with a relatively longer size and higher coverage was obtained after reference assembly, it was used to search the broad phage metagenome database again. This procedure was repeated until the assembled sequence stopped extending. All of the sequence assemblies were generated using the Geneious Pro (version 5.6.2; Biomatters Ltd.). A schematic presentation of the sequence assembly procedure is shown in Figure 1.

### Sequence analysis

The nine putative archaeal RNA virus sequences were searched against the NCBI nucleotide database using BLASTN (E-value < 10^−5^) and against the NCBI non-redundant protein database using BLASTX (E-value < 10^−3^) for the potential homologous sequences in the databases. The REPuter program (Kurtz et al. 2001) was used to identify the repeat sequences.

### Phylogenetic analysis

A conserved genomic fragment of 464 nt was identified in contigs 1, 3 and 4; JQ756122; and JQ756123 by sequence alignment using Geneious Pro (version 5.6.2) and used to reconstruct the phylogenetic trees. Maximum likelihood analyses were performed using phyML (Guindon et al. 2010) with the HKY85 model and 1,000 replicates.

### Nucleotide sequence accession numbers

The nucleotide sequences of the nine contigs were deposited in DDBJ under the accession numbers AB979436 - AB979444.

## Results

**Figure 2:**
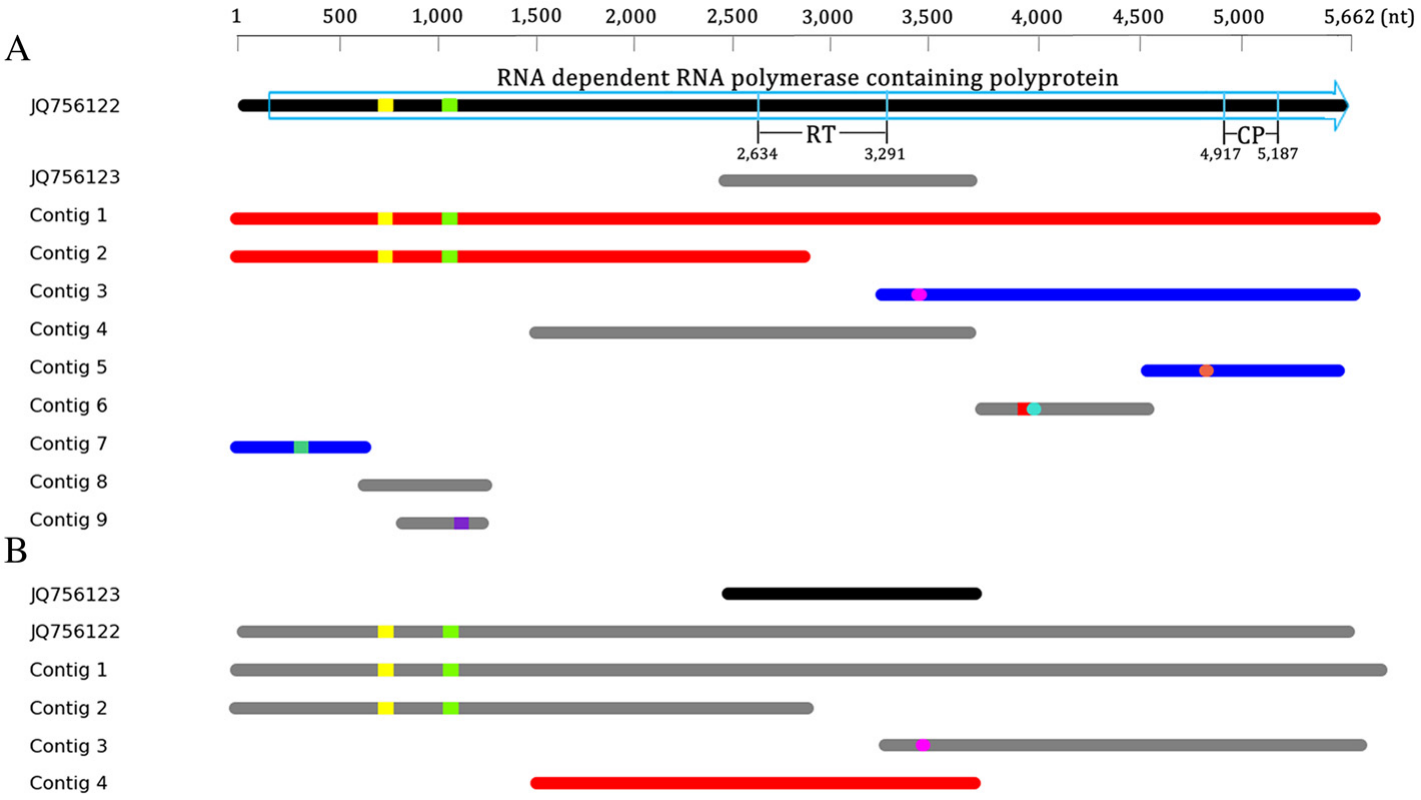
Schematic illustration of sequence similarity (Red, 90-100%; blue, 70-90%; and gray 50-70%) between the 9 contigs and JQ756122 **(A)** / JQ756123 **(B)** and the alignment position. The squares represent reverse repeat sequences, while the dots represent palindromic sequences. Repeat sequences of the same color represent the same repeat sequences. The RNA dependent RNA polymerase gene is labeled with arrow box. RT, reverse transcriptase_like family domain; CP, capsid protein domain described in Bolduc et al..

After the de novo and reference assemblies, nine archaeal RNA-virus-related contigs were obtained. The data regarding the metagenomic assembly of these nine contigs are provided in Table 1. The longest contig was 5,866 nt in length, being longer than the JQ756122 sequence (5,662 nt) by approximately 40 nt at the 5’ end and 170 nt at the 3’ end, while the remaining length was almost identical to the JQ756122 sequence with only a 4-nt difference. The G + C contents of these nine contigs ranged from 49.6 to 54.9% and were very similar to that of the putative archaeal RNA viruses (JQ756122 and JQ756123), whose G + C contents were 50.7 and 52.2%, respectively. A pairwise sequence similarity comparison indicated that the assembled contigs in this study shared a similarity of 50 to 99% with JQ756122 or JQ756123 (Figure 2), suggesting the genetic diversity of the putative archaeal RNA viruses in the Yellowstone hot spring. In total, five reverse-repeat and three palindromic sequences were identified from the nucleotide sequences of 7 contigs and of a putative archaeal RNA virus (JQ756122) using the REPuter program (Table 2) and checked manually. JQ756122 and contigs 1 and 2 shared two types of reverse-repeat sequences (Figure 2) with >97% of sequence similarity. All of the repeat sequences were searched against (BLASTN, E-value < 0.1) the virus database but without a significant hit. The functions of these repeat sequences remain unknown.

**Table 1:** **Data on the metagenomic assembly of nine novel genomic sequences of putative archaeal RNA viruses**

**Contig**	**Length (nt)**	**No. of reads recruited to each genome**	**Identical sites**	**Pair wise identity (%)**	**Coverage**			**G + C content (%)**
					**Mean**	**Minimum**	**Maximum**	
1	5,866	3,273	5,344	98.4	195.5	8	463	50.6
2	2,929	1,437	2,551	97.7	169.2	6	361	50.5
3	2,439	142	2,397	98.8	21.5	2	45	49.6
4	2,241	99	2,202	98.6	16.5	2	40	52.0
5	986	17	970	97.3	5.8	2	13	55.1
6	863	20	851	98.7	8.1	2	16	53.5
7	663	72	647	97.9	36.4	7	62	49.6
8	631	11	529	99.0	6.6	1	11	50.4
9	417	4	315	99.2	3.3	1	4	54.9

**Table 2:** **Repeat sequences in the genomic sequences of putative archaeal RNA viruses**

**Contig**	**Repeat type**	**Length (nt)**	**Repeat position and sequence (5’-3’)**	**E-value**
JQ756122	R1	16		2.10e-3
	R2	15		8.40e-3
1	R1	16		2.25e-3
	R2	15		9.01e-3
2	R1	16		5.62e-4
	R2	15		2.25e-3
3	P1	14		6.23e-3
5	P2	14		1.02e-3
6	P3	14		7.80e-4
	R3	13		3.12e-3
7	R4	18		1.80e-6
9	R5	14		1.82e-4

R represents reverse repeat sequences. P represents palindromic repeat sequences. The arrows indicate repeat units.

BLASTN (E-value < 10^−5^) and BLASTX (E-value < 10^-3)^ analyses showed that all 9 contigs were significantly similar to the sequences of the putative archaeal RNA viruses (JQ756122 or JQ756123) (Additional file 3: Table S3 and Additional file 4: Table S4). These results further confirm that these contigs are the partial or complete genomes of putative novel archaeal RNA virus isolates that are closely or distantly related to the reported isolates (Bolduc et al. 2012).

**Figure 3:**
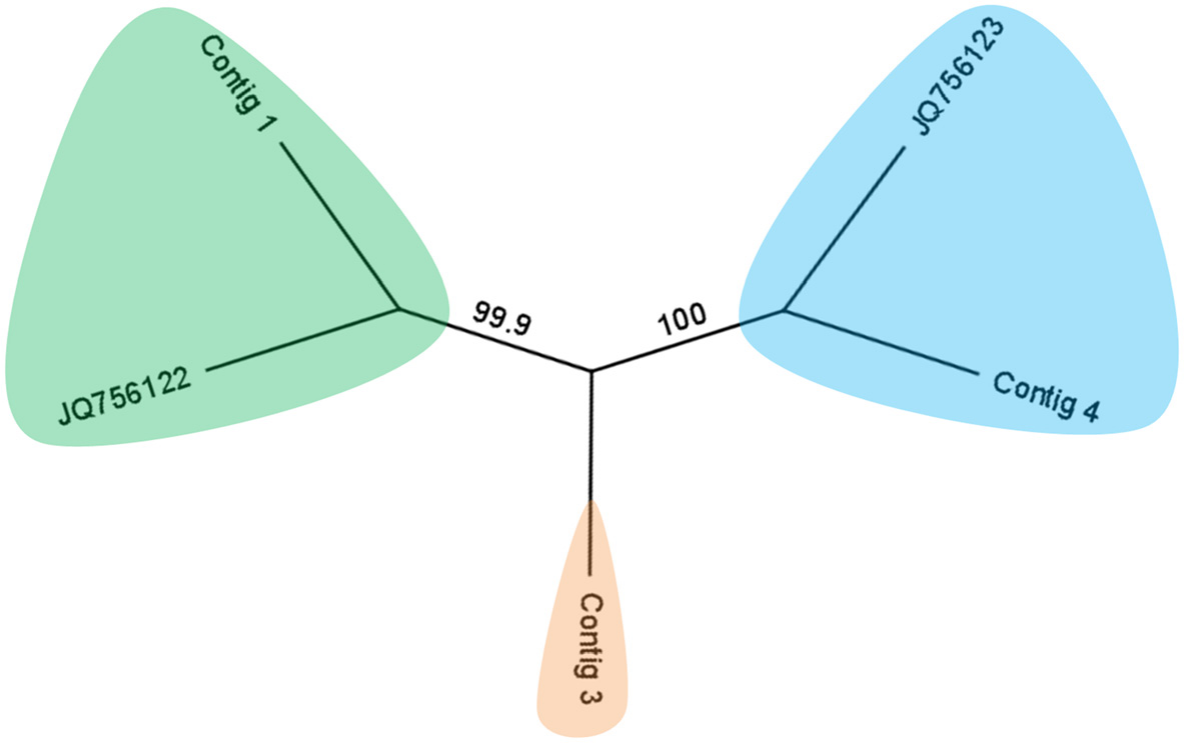
Unrooted phylogenetic tree (maximum likelihood; model: HKY85; 1000 bootstrap replicates) showing 3 lineages of the putative archaeal RNA viruses as marked in different background colors.

Phylogenetic analyses indicate 3 lineages of the putative archaeal RNA viruses (Figure 3); contig 1 was closely related to JQ756122, and contig 4 was closely related to JQ756123. Contig 3 represented the third genogroup. Given the relatively low sequence similarity between other the contigs and JQ756122 or JQ756123, it is reasonable to speculate that putative archaeal RNA viruses are genetically diverse in the Yellowstone hot spring.

## Discussion

To investigate the worldwide diversity of the putative archaeal RNA viruses, the nucleotide sequence JQ756122 was used to search against global metagenomic databases to retrieve significantly similar reads. Subsequently, based on both the de novo and reference sequence assemblies of these retrieved reads, nine novel partial or nearly complete genomes of the putative archaeal RNA viruses were successfully obtained. Similar mapping methods have been used by our group to assemble the genomic sequences of novel virophages in the CAMERA metagenomic datasets, through which seven complete virophage genomic sequences were obtained (Zhou et al. 2013; Zhou et al. 2015). Consequently, the established sequence assembly procedures generate a better understanding of the genetic diversity of enigmatic viruses and can be applied to similar studies.

Interestingly, all 3,757 of the putative archaeal RNA virus-related RNA-origin sequences were detected in the metagenomic dataset of sample NL10 (GPS coordinate: N44.7535, W-110.7238) collected by Bolduc et al. (Bolduc et al. 2012) in the acidic hot spring in Yellowstone National Park. It indicates that the associated archaeal RNA viruses may be unique to this location. Similar archaeal RNA viruses may also exist in other environments. The absence of related reads in other metagenomic datasets may result from the relatively small number of RNA metagenomic datasets compared to the number of DNA metagenomic datasets. In addition, other environments may also possess archaeal RNA viruses whose genomes are quite different from the putative archaeal RNA viruses that were identified in Yellowstone National Park. The genome sequencing of archaeal viruses has revealed very few genes whose products have significant sequence similarity to any known proteins (Prangishvili et al. 2006; Pina et al. 2011), and only a few homologous genes are shared between the members of different families of crenarchaeal viruses (Prangishvili 2013). Accordingly, archaeal RNA viruses in different or even in the same environment may have different genome contents.

Bolduc et al. identified CRISPRs from cellular metagenomes (Bolduc et al. 2012). Direct repeats and spacers were extracted from the identified CRISPRs, and the CRISPR spacers were then compared against the viral RNA metagenome. In their paper, these authors reported that “Forty-six spacers, associated with 4 types of direct repeats, were identical to RNA sequences within the viral metagenome. The majority of matching spacer sequences of the RNA metagenome (44/46) were related to DRs of the archaeal species *Sulfolobus islandicus* and *Sulfolobus acidocaldarius*. These findings suggest that the RNA viral genomes replicate in an archaeal host belonging to the *Sulfolobales*, a cell type commonly found in NL10 and acidic hot springs worldwide, and elicit a CRISPR-mediated immune response.” These 4 types of direct repeats were searched here against nine contigs. However, no identical matches were observed. These 4 types of direct repeats were also absent in the two contigs that were assembled by Bolduc et al. Therefore, we could not determine whether the potential host of the nine contigs here is *Sulfolobus*. However, Bolduc et al. demonstrated that the potential host of their two contigs was archaea. Stedman et al. argued that the host of the putative archaeal RNA viruses that were identified by Bolduc et al. is not archaea and may be a novel phylogenetic lineage based on the fact that the codon usage frequencies of the two contigs from Bolduc et al. are very different from that of the claimed host (Stedman et al. 2013). However, there are numerous examples of virus codon usage either matching or significantly deviating from their host cell codon usage (Young et al. 2013). Additional evidence from Bolduc et al. demonstrating that the origin of the host of two contigs that were assembled by these authors is putative archaea and the fact that the nine contigs here showed significant similarities to the two contigs of Bolduc et al. indirectly demonstrate that these nine contigs are putative archaeal RNA viral sequences.

Bolduc et al. identify two genomic fragments of the putative archaeal RNA viruses (Bolduc et al. 2012). In this study, we find 9 assembled sequences that are related to the putative archaeal RNA viruses. Each sequence represents one possible novel viral genogroup or genotype. At least three viral lineages were observed phylogenetically, indicating that putative archaeal RNA viruses are genetically diverse in the acidic hot springs and that archaeal RNA viruses may have great diversity in light of the diversity and number of archaeal hosts in the environment being the same as that of the viruses of Bacteria and Eukarya.

Thus far, little is known about the biological features of archaeal RNA viruses. Whether such viruses exist in the environment requires further study via isolation and identification. However, based on these available sequences, specific primers can be designed to survey the distribution, diversity and dynamics of these putative archaeal RNA viruses in various interesting environments. In addition, additional metagenomic sequencing work needs to be performed, which would contribute greatly to the discovery of novel archaeal RNA viruses, which in turn would provide additional insight into the diversity, evolution and ecology of archaeal RNA viruses and their hosts.

## Additional files

Additional file 1: Table S1. Data sets in the CAMERA containing reads significantly similar to that of the putative archaeal RNA viruses. Additional file 2: Table S2. Sample information of these 3,757 RNA- and 6 DNA-origin reads retrieved from the CAMERA 2.0 Portal. Additional file 3: Table S3. BLASTN results of the nine contigs (E-value < 10^-5^). Additional file 4: Table S4. BLASTX results of the nine contigs (E-value < 10^-3^).
