# Supplementary material for: Diversity of putative archaeal RNA viruses in metagenomic datasets of a yellowstone acidic hot spring
Source: Springerplus. 2015 Apr 18;4:189. doi: 10.1186/s40064-015-0973-z (PMC4405519; doi:10.1186/s40064-015-0973-z)
Supplement: Additional file 1: Table S1. — Data sets in the CAMERA containing reads significantly similar to that of the putative archaeal RNA viruses. [file 40064_2015_973_MOESM1_ESM.doc]

**Table S1.** Data sets in the CAMERA containing reads significantly similar to that of the putative archaeal RNA viruses

| **Data set** | **no. of matched reads** |
| --- | --- |
| Broadphage metagenomes: all metagenomic 454 reads (N) | 3,763 |
| All metagenomic 454 whole genome shotgun reads (N) | 3,736 |
| All metagenomic 454 reads (N) | 3,730 |
| NCBI Environmental Sample Nucleotides | 1 |
