# Supplementary material for: Diversity of putative archaeal RNA viruses in metagenomic datasets of a yellowstone acidic hot spring
Source: Springerplus. 2015 Apr 18;4:189. doi: 10.1186/s40064-015-0973-z (PMC4405519; doi:10.1186/s40064-015-0973-z)
Supplement: Additional file 2: Table S2. — Sample information of these 3,757 RNA- and 6 DNA-origin reads retrieved from the CAMERA 2.0 Portal. [file 40064_2015_973_MOESM2_ESM.doc]

**Table S2.** Sample information of these 3,757 RNA- and 6 DNA-origin reads retrieved from the CAMERA 2.0 Portal

|  | RNA-origin | | DNA-origin |
| --- | --- | --- | --- |
|  | Multiple hosts NL 10_2010-02 RNA (3755) | Multiple hosts WTA NL 16 (2) | Viral metagenome RIFCO pool (6) |
| Site Id | CAM_BM_000953 | CAM_BM_000957 | CAM_BM_001230 |
| Project | CAM_PROJ_BroadPhage | CAM_PROJ_BroadPhage | CAM_PROJ_BroadPhage |
| Location | Yellowstone National Park | Yellowstone National Park | Rifle DOE IFRC, Rifle Colorado |
| Region | Yellowstone National Park | Yellowstone National Park | Rifle DOE IFRC, Rifle Colorado |
| Comment | Acidic Hot Spring | Acidic Hot Spring | Upgradient well 2 |
| Latitude | 44.7535 | 44.7535 | 39.53 |
| Longitude | -110.7238 | -110.7238 | -107.73 |
| Habitat | Acidic, thermophilic hot spring | Acidic, thermophilic hot spring | Groundwater |
| Date collected | 02/19/10 | 10/09/09 | 08/01/08 |
| pH | 3 | 3 | 7 |
| Temperature (℃) | 83 | 83 | 10 |
| Volume (L) | 1.2 | 1.2 |  |
| Filter size | null - 0.2 µm | null - 0.2 µm | null - 0.22 µm |
