# Supplementary material for: Diversity of putative archaeal RNA viruses in metagenomic datasets of a yellowstone acidic hot spring
Source: Springerplus. 2015 Apr 18;4:189. doi: 10.1186/s40064-015-0973-z (PMC4405519; doi:10.1186/s40064-015-0973-z)
Supplement: Additional file 3: Table S3. — BLASTN results of the nine contigs (E-value < 10-5). [file 40064_2015_973_MOESM3_ESM.doc]

**Table S3.** BLASTN results of the nine contigs (E-value < 10^-5^)

| Contig | Length (nt) | Best hits in GeneBank nr database | | | | |
| --- | --- | --- | --- | --- | --- | --- |
|  |  | **Gene, species** | **Accession** | **nt identity %** | **E-value** | **Alignment length in**  **nt (start-end position)** |
| 1 | 5,866 | RNA-dependent RNA polymerase gene, partial cds;  Uncultured virus clone contig00002 | JQ756122.1 | 99 | 0.0 | 5,663 (39-5,699) |
|  |  | RNA-dependent RNA polymerase gene, complete cds;  Uncultured virus clone contig00228 | JQ756123.1 | 72 | 2e-155 | 1,125 (2,632-3,746) |
| 2 | 2,929 | RNA-dependent RNA polymerase gene, partial cds;  Uncultured virus clone contig00002 | JQ756122.1 | 97 | 0.0 | 2,889 (40-2,926) |
|  |  | RNA-dependent RNA polymerase gene, complete cds;  Uncultured virus clone contig00228 | JQ756123.1 | 73 | 6e-42 | 347 (2,558-2,902) |
| 3 | 2,439 | RNA-dependent RNA polymerase gene, partial cds;  Uncultured virus clone contig00002 | JQ756122.1 | 71 | 0.0 | 2,388 (3-2,375) |
|  |  | RNA-dependent RNA polymerase gene, complete cds;  Uncultured virus clone contig00228 | JQ756123.1 | 68 | 6e-23 | 399 (1-396) |
| 4 | 2,241 | RNA-dependent RNA polymerase gene, complete cds;  Uncultured virus clone contig00228 | JQ756123.1 | 99 | 0.0 | 1,268 (978-2,241) |
|  |  | RNA-dependent RNA polymerase gene, partial cds;  Uncultured virus clone contig00002 | JQ756122.1 | 70  71 | 0.0  1e-44 | 1,487 (732-2,204)  394 (1-393) |
| 5 | 986 | RNA-dependent RNA polymerase gene, partial cds;  Uncultured virus clone contig00002 | JQ756122.1 | 73 | 2e-156 | 971 (13-978) |
| 6 | 863 | RNA-dependent RNA polymerase gene, partial cds;  Uncultured virus clone contig00002 | JQ756122.1 | 73 | 3e-96 | 617 (229-843) |
| 7 | 663 | RNA-dependent RNA polymerase gene, partial cds;  Uncultured virus clone contig00002 | JQ756122.1 | 89 | 2e-111 | 314 (40-353) |
| 8 | 631 | RNA-dependent RNA polymerase gene, partial cds;  Uncultured virus clone contig00002 | JQ756122.1 | 66 | 2e-09 | 401 (61-452) |
