# Supplementary material for: Diversity of putative archaeal RNA viruses in metagenomic datasets of a yellowstone acidic hot spring
Source: Springerplus. 2015 Apr 18;4:189. doi: 10.1186/s40064-015-0973-z (PMC4405519; doi:10.1186/s40064-015-0973-z)
Supplement: Additional file 4: Table S4. — BLASTX results of the nine contigs (E-value < 10-3). [file 40064_2015_973_MOESM4_ESM.doc]

**Table S4.** BLASTX results of the nine contigs (E-value < 10^-3^)

| Contig | Length (nt) | Best hits in GeneBank nr database | | | | | |
| --- | --- | --- | --- | --- | --- | --- | --- |
|  |  | **Protein, species** | **Accession** | **aa identity %** | **E-value** | **Alignment length in aa (start-end position)** | **Original query frame** |
| 1 | 5,866 | RNA-dependent RNA polymerase, partial  [uncultured virus] | AFE55712.1 | 99 | 0 | 832 (272-2,767) | +2 |
|  |  | RNA-dependent RNA polymerase, partial  [uncultured virus] | AFE55712.1 | 99 | 0 | 703 (2,761-4,869) | +1 |
|  |  | RNA-dependent RNA polymerase, partial  [uncultured virus] | AFE55712.1 | 99 | 2e-117 | 275 (4,854-5,678) | +3 |
|  |  | RNA-dependent RNA polymerase  [uncultured virus] | AFE55713.1 | 82 | 2e-103 | 45 (2,633-2,767) | +2 |
|  |  | RNA-dependent RNA polymerase  [uncultured virus] | AFE55713.1 | 80 | 2e-103 | 182 (2,761-3,306) | +1 |
|  |  | RNA-dependent RNA polymerase  [uncultured virus] | AFE55713.1 | 43 | 2e-103 | 28 (3,303-3,386) | +3 |
| 2 | 2,929 | RNA-dependent RNA polymerase, partial  [uncultured virus] | AFE55712.1 | 98 | 0 | 885 (273-2,927) | +3 |
|  |  | RNA-dependent RNA polymerase  [uncultured virus] | AFE55713.1 | 77 | 1e-41 | 98 (2,634-2,927) | +3 |
| 3 | 2,439 | RNA-dependent RNA polymerase, partial  [uncultured virus] | AFE55712.1 | 87 | 0 | 258 (1,600-2,373) | +1 |
|  |  | RNA-dependent RNA polymerase, partial  [uncultured virus] | AFE55712.1 | 71 | 0 | 403 (3-1,211) | +3 |
|  |  | RNA-dependent RNA polymerase, partial  [uncultured virus] | AFE55712.1 | 90 | 0 | 133 (1,211-1,609) | +2 |
| 4 | 2,241 | RNA-dependent RNA polymerase, partial  [uncultured virus] | AFE55712.1 | 74 | 0 | 241 (1,492-2,214) | +1 |
|  |  | RNA-dependent RNA polymerase, partial  [uncultured virus] | AFE55712.1 | 68 | 0 | 240 (693-1,412) | +3 |
|  |  | RNA-dependent RNA polymerase, partial  [uncultured virus] | AFE55712.1 | 81 | 0 | 27 (1,409-1,489) | +2 |
|  |  | RNA-dependent RNA polymerase, partial  [uncultured virus] | AFE55712.1 | 63 | 4e-65 | 243 (2-730) | +2 |
|  |  | RNA-dependent RNA polymerase  [uncultured virus] | AFE55713.1 | 100 | 4e-132 | 109 (1,086-1,412) | +3 |
|  |  | RNA-dependent RNA polymerase  [uncultured virus] | AFE55713.1 | 100 | 4e-132 | 27 (1,409-1,489) | +2 |
|  |  | RNA-dependent RNA polymerase  [uncultured virus] | AFE55713.1 | 97 | 4e-132 | 95 (1,489-1,773) | +1 |
|  |  | RNA-dependent RNA polymerase  [uncultured virus] | AFE55713.1 | 100 | 4e-132 | 28 (1,761-1,844) | +3 |
| 5 | 986 | RNA-dependent RNA polymerase, partial  [uncultured virus] | AFE55712.1 | 70 | 4e-102 | 329 (7-984) | +1 |
|  |  | RNA-dependent RNA polymerase, partial  [uncultured virus] | AFE55712.1 | 70 | 1e-16 | 64 (239-427) | +2 |
| 6 | 863 | RNA-dependent RNA polymerase, partial  [uncultured virus] | AFE55712.1 | 81 | 1e-112 | 214 (206-847) | +2 |
| 7 | 663 | RNA-dependent RNA polymerase, partial  [uncultured virus] | AFE55712.1 | 52 | 1e-22 | 114 (274-615) | +1 |
| 8 | 631 | RNA-dependent RNA polymerase, partial  [uncultured virus] | AFE55712.1 | 58 | 6e-55 | 190 (60-626) | +3 |
| 9 | 417 | RNA-dependent RNA polymerase, partial  [uncultured virus] | AFE55712.1 | 41 | 3e-19 | 135 (1-399) | +1 |
